# Current State of the Diagnosis of Invasive Pulmonary Aspergillosis in Lung Transplantation

**DOI:** 10.3389/fmicb.2018.03273

**Published:** 2019-01-09

**Authors:** Sabina Herrera, Shahid Husain

**Affiliations:** Transplant Infectious Diseases, Toronto General Hospital, University Health Network, University of Toronto, Toronto, ON, Canada

**Keywords:** lung transplant, invasive aspergillosis, PCR, *Aspergillus*, immunocompromized, galactomannan, BDglucan, cytokines

## Abstract

As the number of lung transplants performed worldwide each year continues to grow, the success of this procedure is threatened by the incidence of non-CMV infections such as invasive aspergillosis. Despite tremendous efforts and the availability of numerous diagnostic tests (especially in hematological malignancies) the diagnosis of invasive aspergillosis continues to be a challenge. Lung transplantation remains a unique clinical scenario, where additional host defenses are immunocompromized, making many of the available tests unsuitable. In this review we will navigate through the myriad of diagnostic tests currently available and how they apply to this unique patient population, as well as have a look into what the future holds.

## Introduction

Lung transplantation is an established modality for end-stage pulmonary disease. With over 4600 lung transplants performed in 2016 worldwide (Lund et al., 2017) the number of these procedures carried out annually continues to grow. One of the greatest challenges for the survival of these patients during the first year after transplant are non-CMV infections, especially invasive pulmonary aspergillosis (IPA), which portends a higher mortality than bacterial or viral infections (Mattner et al., 2007).

Several strategies are being used to prevent the development of IPA including universal and pre-emptive prophylaxis (Husain et al., 2016). However, the diagnosis of IPA in lung transplantation remains challenging. *Aspergillus* is a ubiquitous organism, and is often found in sputum or bronchoalveolar lavage (BAL) samples. The presence of *Aspergillus* in these respiratory samples does not necessarily represent a true infection, but in most cases is merely indicative of colonization. Nevertheless, colonization itself is not a risk factor to be overlooked. In the context of immunosuppression and decreased mucociliary motility, colonization and other risk factors can often lead to a true invasive infection; highlighting the importance of having precise diagnostic tools. Moreover, diagnostic tests used in other patient populations such as hematological malignancies cannot be readily applied in Lung transplant recipients (LTRs).

In this paper we will review the current state of the diagnosis of invasive aspergillosis in lung transplantation, and will comment on the currently available tests as well as diagnostic tests in development, that will available in the near future.

## Diagnosis of Invasive Pulmonary Aspergillosis in Lung Transplant Recipients

Invasive pulmonary aspergillosis in LTRs has several particularities that are not accounted for in The European Organisation for Research and Treatment of Cancer (EORTC) guidelines, such as colonization, tracheobronchitis and bronchial anastomotic infections. It is therefore preferred to use the International Society for Heart and Lung Transplantation (ISHLT) guidelines (Husain et al., 2011) for the diagnosis of these patients, as it includes diagnostic criteria for all these entities. For the diagnosis of ‘proven IPA’ there must be a biopsy showing histologic evidence of parenchymal invasion by fungal hyphae, or pseudohyphae, or positive culture from sterile tissue *ALONE*; *OR* with sign/symptoms AND radiological criteria AND laboratory criteria [single positive culture for mold BAL/blood *OR* single positive PCR for mold BAL/blood *OR* positive galactomannan in the BAL; *OR* at least TWO positive sputum cultures/PCRs of fungal organisms (excluding *Candida* species)]. For ‘probable IPA’ their criteria are the same as for proven, except there is negative or absent histology.

## Microbiology and Microscopy

There have been immense advances in the past decade in the development of molecular and immunological tests for the diagnosis of IPA. However, these are not widely available or standardized, and culture and microscopy still play an important role in the diagnosis of IPA. Microscopic methods such as wet mounts, Gram stains, and conventional histopathology, are useful suggesting the presence of *Aspergillus* spp. in parenchyma biopsies or BAL samples for the diagnosis of IPA (McClenny, 2005). *Aspergillus* spp. grows as septate hyphae 2.5–4.5 μm in diameter and can be characterized as branching dichotomously (∼ 45°C angle) (Lass-Flörl and Mayr, 2009). Demonstration of *Aspergillus* spp. in tissue is the only way to prove IPA, however, a negative result cannot rule it out. For a definite identification of the species, a culture or the use of molecular methods is required, in addition to histology. *Aspergillus* spp. can grow from fungal media and also from sheep blood agar commonly used for bacterial culture. Culture has the advantage of speciation and ability to perform susceptibility testing. Their sensitivity nevertheless is far from ideal with a positive culture in sputum in 8 to 34% of patients with IPA and 45 to 62% in BAL (Horvath and Dummer, 1996; Hoenigl et al., 2014). Sensitivity is significantly higher in samples from lung biopsies (Lass-Flörl et al., 2007). The main caveat for these methods are the processing times, especially in immunocompromized patients where diagnosis is truly time-sensitive. Additionally in LTRs culture is unable to differentiate colonization from invasive disease, unless the culture is done directly from a tissue sample. Since *Aspergillus* spp. is ubiquitous, contamination of samples can also occur. Trained staff in the lab are essential for better performance of the aforementioned tests.

## Serological and Molecular Tests

Serological tests are among the most widely used tests for the diagnosis of IPA. This is in part the result of the poor yield of respiratory sample cultures.

### *Aspergillus* Galactomannan

The major antigenic component secreted by *A*. *fumigatus* into the growth medium is galactomannan (GM) – a soluble polysaccharide that is present in the cell wall of most *Aspergillus* and *Penicillium* species (Latge et al., 1994). We can detect GM in biological fluids by a commercial sandwich enzyme-linked immunosorbent assay (ELISA) such as the Platelia^TM^ *Aspergillus* (Bio-Rad, Marnes-la-Coquette, France) (Stynen et al., 1995). Despite being used extensively, the ELISA lacks some of the advantages that cultures provide; notably speciation and sensitivities. Therefore, it is generally used in combination with cultures or histological samples. In LTRs GM has been studied both in serum and in BAL, having a better performance in the latter (Clancy et al., 2007). It’s sensitivity and specificity range from 60 to 100% and 85 to 98%, respectively, according to different studies including several meta-analysis (Pfeiffer et al., 2006; Clancy et al., 2007; Husain et al., 2007, 2008; Zou et al., 2012; see Table 1). It is to note that some of the studies were not done purely on LTR and some included other SOT and some hematological malignancies (Pfeiffer et al., 2006; Zou et al., 2012).

**Table 1:** Summary of human studies assessing galactomannan, BD-glucan and *Aspergillus* PRC in lung transplantation.

Study	Number patients recruited	Sample	Sensitivity	Specificity	Outcome
**GM**					
Bhimji et al., 2018	197 LTR	EBC			GM detectable in EBC but no correlation with IA
Zou et al., 2012	3344 Patients or controls (614 with IPA)	BAL	87%	89%	Meta-analysis including 30 studies until 2012, mainly hematological but also SOT
Pasqualotto et al., 2010	60 LTRs, 8 with probable or proven IPA	BAL	100%	40%	Increasing the cutoff to 1.5 improved the specificity (90.4%) maintaining sensitivity
Husain et al., 2008	11 Patients and 185 controls (119 LTRs)	BAL	81.8%	95.8%	Higher specificity with cut-off of 1.0 (96.6%)
Clancy et al., 2007	5 Patients with IPA and 76 controls (16 LTRs)	BAL	100%	90.8%	The sensitivity of BAL GM testing was better than serum GM or BAL cytology and culture. Increasing the cutoff to ≥1 improved the specificity (90.8%)
Husain et al., 2007	116 LTR, 6 with probable or proven IPA	BAL	60%	95%	Increasing the index cutoff value to 1.0 or more yielded a sensitivity of 60%, a specificity of 98%
Pfeiffer et al., 2006		Serum	71%	85%	Meta-analysis including 27 studies from 1996 to 2005, mainly hematological but also SOT
**BD glucan**					
Bhaskaran et al., 2017	195 Samples LTR, and 10 episodes of IPA	BAL	80%	53%	Using 41 pg/ml as cut-off, sensitivity and specificity improved to 75 and 91%, respectively, at a 524 pg/ml cut-off
Mutschlechner et al., 2015	135 SOT patients including LTR	BAL and serum	79.2% 79.2%	38.5% 81.8%	233 BAL and 109 serum specimens. Multicenter studies. 135 SOT patients, 114 LTRs. Based on a 100-pg/ml positive cutoff
Alexander et al., 2010	14 LTRs with proven or probable IFI and 59 LTRs controls	Serum	71%	59%	756 Specimens from 59 subjects without IFI and 41 specimens from 14 patients with proven or probable IFI. Based on a 60-pg/ml positive cutoff
**PCR**					
Lass-Flörl et al., 2011	9 SOT and 33 patients with HM	Lung and skin	82%	79%	Sensitivity of the MycAssay *Aspergillus* test was 82% and specificity 79% relative to microscopy and 90 and 64%, respectively, compared with *Aspergillus* culture
Luong et al., 2011	150 LTRs (16 proven/probable IPA, 26 colonized, 11 non-*Aspergillus* mold colonization, and 97 negative controls)	BAL	100%	88%	The sensitivity and specificity of *A. fumigatus*-specific PCR were 85 and 96%, respectively

*BAL, bronchoalveolar lavage; EBC, exhaled breath condensate; GM, galactomannan; HM, hematological malignancy; IFI, invasive fungal infection; IPA, invasive pulmonary aspergillosis; LTR, lung transplant recipient; PCR, polymerase chain reaction; SOT, solid organ transplant*.

Although an optical density cut-off of 0.5 is generally used in both serum and BAL assays, several of these studies showed that increasing the optical density cut-off to 1.0 in BAL increased the specificity of the assay (Clancy et al., 2007; Husain et al., 2007, 2008). Husain et al. (2007) studied 116 LTRs, 6 with probable or proven IPA, finding sensitivity of 60% and specificity of 95%. By increasing the index cutoff value for BAL to >0.66 or = 1.0 a specificity of 98% was achieved, while maintaining a sensitivity of 60%. Similar findings were reported by Clancy et al. (2007) who noted that increasing the cut-off to ≥1 improved the specificity to 90.8%, and by Husain et al. (2008), who demonstrated a higher specificity of 96.6% with cut-off of 1.0 in a larger cohort of LTRs.

As the *Aspergillus* GM assay is a widely used tool, it is often used to monitor therapeutic response in LTRs. In practice, serum GM is more feasible for monitoring than BAL GM, as it does not require the repeated bronchoscopies necessary to obtain multiple BAL samples. However, GM has several limitations that need to be taken into account. Firstly, a positive BAL GM does not necessarily mean there is an invasive disease as a positive BAL GM result can also be found in colonized patients (Herrera and Husain, 2018). Secondly, both false positive and false negative results have been reported with varying frequency. Cross reactivity has been reported in patients with *Paracoccidioides brasiliensis*, *Histoplasma capsulatum*, and *Cryptococcus* (Xavier et al., 2009). Use of Piperacillin-tazobactam has also been associated with false positive GM in the past (Sulahian et al., 2003), as its production is the result of the fermentation product of molds of the genus *Penicillium* that also contain GM in their wall (Aubry et al., 2006). This issue seems to be less of a problem recently with new formulations of Piperacillin-tazobactam (Vergidis et al., 2014). In children, it has been hypothesized that GM present in some food (such as milk, rice, or protein nutriments) can possibly pass through the intestinal mucosa and in turn actuate a false positive result (Siemann et al., 1998; Gangneux et al., 2002). Finally, Plasma-Lyte an electrolyte replacement solution containing sodium gluconate, has also been identified as a cause of false positive GM in BAL samples (Muñoz et al., 2003).

In LTRs, false positivity of the GM assay was reported in 20% of the patients in a 2004 study by Husain et al. (2004) Importantly, most false-positive tests occurred in the early post-transplant period, with 79% occurring in the first 14 days following transplantation. This is a limitation of the assay as it is the time period where patients at are at higher immunosuppressive risk, and a false positive result would prompt the physicians into initiating unnecessary treatment. This phenomenon was more likely to happen in patients with the underlying diseases of cystic fibrosis (CF) or chronic obstructive pulmonary disease (COPD), as these patient populations are known to have higher rates of *Aspergillus* colonization. Pasqualotto et al. (2010) also found a high rate of false positivity, in a 2010 study 55.5% of the cases were associated with *Aspergillus* colonization when using a cut-off of 1.0. False positivity was also noted to be higher in single lung transplant recipients.

On the other hand, patients receiving anti-mold prophylaxis or treatment often have false negative GM results (Duarte et al., 2014). Other causes of false negative results include patients who have been in contact with *Aspergillus* before, as they may develop antibodies, which reduces the GM available for the assay to detect (Mennink-Kersten et al., 2004). Both situations are common scenarios after lung transplantation and may reduce the sensitivity of the assay. A previous study in 2013 also suggested that the GM optical index cut-off might vary according to the species of *Aspergillus* spp. (Xavier et al., 2013).

### (1-3)-β-d-Glucan

(1-3)-β-d-glucan is another cell wall polysaccharide that is found in most fungi, with the exception of *Cryptococcus, Zygomycetes*, and *Blastomyces dermatitidis*. Several (1-3)-β-d-glucan assays have been developed by different companies and use different cut-offs: Fungitell 60–80 pg/mL (Associates of Cape Cod, East Falmouth, MA, United States), Endosafe-PTS 10–1000 pg/mL (Charles River Laboratories, Charleston, SC), Fungitec-G 20 pg/mL (Seikagaku Biobusiness, Tokyo, Japan), beta-Glucan Test 11 pg/mL (Waco Pure Chemical Industries, Osaka, Japan), and BGSTAR β-Glucan Test 11 pg/mL (Maruha, Tokyo, Japan). It is a chromogenic kinetic assay based on the *Limulus* test. To summarize, β-Glucan (BG) activates factor G, a protease of *Limulus* amebocyte lysate (LAL), which is extracted from the amebocytes of horseshoe crab species. This triggers the activation of a coagulation cascade, and the activity of this reaction is measured (Hope et al., 2005).

The *Limulus* test has been used for the diagnosis of numerous invasive fungal infections, including IPA. Its performance has been shown to be best in hematological malignancies (Senn et al., 2008). In solid organ transplantation and lung transplant in particular, it has several limitations that have lead GM to be a better choice for the diagnosis of IPA. The studies performed, in both serum and BAL in LTRs have shown poor results, with sensitivities and specificities ranging from 71 to 80% and 38 to 81%, respectively (Alexander et al., 2010; Mutschlechner et al., 2015; Bhaskaran et al., 2017; Table 1). One study achieved an increased sensitivity and specificity in LTR’s by increasing the cut-off of the Fungitell assay to 524 pg/ml (Bhaskaran et al., 2017), a nearly sevenfold increase from the cut-off of 40–80 pg/ml used as standard. All studies have consistent results showing a very low specificity, which limits the use of this assay for IPA diagnosis. Conversely, it has in fact been used in other invasive fungal infections, such as Candida infections (Ahmed et al., 2017) and *Pneumocystis jirovecii* pneumonia, where it has an excellent performance according to a recent meta-analysis (Karageorgopoulos et al., 2013).

Other issues that limit the use of this assay include false positive results due to cross reactions with certain hemodialysis filters, beta-lactam antimicrobials, and immunoglobulins (Theel and Doern, 2013), all of them frequently used in the lung transplant population. Additionally, it is unable to differentiate colonization from invasive disease in BAL (Herrera and Husain, 2018). Its role in the management of lung transplant recipients is not yet well defined.

### *Aspergillus* PCR

Many polymerase chain reaction (PCR) tests for *Aspergillus* spp. have been developed with great expectations as a promising tool to solve the enigma of IPA diagnosis. However, to date they have failed to live up to those expectations. Several PCRs have been developed according to primer selection (panfungal, genus specific, or species specific), and PCR formats (qualitative, quantitative, and real-time). DNA in respiratory samples of LTRs, has been studied by two standardized *Aspergillus* assays, Viracor (Viracor-IBT Laboratories) and MycAssay (Myconostica). One of the main caveats that this test faces is the lack of standardization in the procurement of samples. Its costs exceed those of GM or β-d-glucan, and it is not available in most centers. Furthermore, as with the aforementioned tests it is unable to differentiate colonization from invasive disease (Herrera and Husain, 2018) and unable to identify subspecies; requiring additional specific subspecies PCR and unable to provide antifungal susceptibilities. Despite these limitations and the lack of studies that have been performed in the LTR population, the few available studies have shown very good outcomes with sensitives and specificities of 100 and 88%, respectively, for diagnosis of IPA; and sensitivity and specificity for *A. fumigatus*-specific PCR of 85 and 96%, respectively (Luong et al., 2011; Table 1). Additional advantages include the ability to identify mutations associated with treatment resistance (Bernal-Martínez et al., 2017; Dannaoui et al., 2017) that are usually time consuming using conventional methods alone. Although AsperGenius (PathoNostics, Maastricht, Netherlands), has not been yet tested in LTRs for the identification of azole resistance in *A. fumigatus*, this could have a broad potential in this population, especially in colonized cystic fibrosis patients undergoing lung transplantation (Chong et al., 2016). Multiple PCR tests are being developed, but unfortunately none of these have been tested in LTRs (Salehi et al., 2016). All of these factors indicate that this test will be a very promising tool in the near future.

Table 1 summarizes the studies available in LTRs for the different serological and molecular markers available.

## Newer Tests

### Monoclonal Antibodies

Use of monoclonal antibodies has changed many fields in medicine over the past decades. Some centers have attempted to develop monoclonal antibodies against *Aspergillus* spp. for the diagnosis of IPA.

The first of these developed was the lateral flow device, an immunochromatographic assay that uses JF5, a monoclonal antibody that binds to an extracellular glycoprotein secreted during active growth of *Aspergillus* spp. This assay has been tested in several populations, including SOT (Willinger et al., 2014). It seems to display a better performance in BAL, where pooled sensitivity and specificity for the proven/probable IPA was assessed in a recent meta-analysis showing 86 and 93%, respectively (Pan et al., 2015). Its main strength is high specificity, and a new version of the assays shows improved specificity compared to the older assay according to a recent study (Hoenigl et al., 2018). However, the noted sensitivity of this assay is not as good, and is further decreased in patients receiving anti-mold prophylaxis (Castillo et al., 2017). Additionally, cross-reactivity has been reported with *Penicillium* spp. (Heldt and Hoenigl, 2017). So far the lateral flow device has only been studied in cohorts including a mix of LTR’s, patients with hematological malignancies, or other solid organ transplants (Castillo et al., 2017). Thus, extrapolating the results for these mixed cohorts may not give an accurate indication of the sensitivity and specificity for LTR’s alone.

Most recently, a group from Baltimore United States used an anti-*A. fumigatus* antibody (mAb476) that rapidly detects a fungal antigen in urine (Marr et al., 2018), avoiding the need for more invasive tests. The use of this antibody test in IPA had previously been proven possible only in mice and guinea pig models (Dufresne et al., 2012). In their human study, they found that the best performance was in patients who had hematological malignancies, whereas the sensitivity from the small subgroup with other underlying diseases was lower at 63.6%. Several false negative results were found in LTRs who had non-invasive forms of the disease. Excluding these patients gave a higher sensitivity of 88.5% for the test. Specificities where high overall, at around 92%. However, false positive results were also found in patients with Histoplasmosis. The advantages offered by this test are, simplicity and the possibility of earlier diagnosis. Nevertheless, further studies are needed in LTRs as with the scarce available data, it seems more advantageous in hematological malignancies.

### Volatile Organic Compounds

A very original test has been able to identify a profile of volatile organic compounds exhaled in the breath of patients with pathogenic *Aspergillus.* These compounds are present during the growth phase of the fungus, in patients undergoing evaluation for IPA. Detection of α-trans-bergamotene, β-trans-bergamotene, a β-vatirenene-like sesquiterpene, or trans-geranylacetone identified IPA patients with 94% sensitivity and 93% specificity (Koo et al., 2014). The study was done in a heterogeneous cohort of immunocompromized patients. In LTRs volatile organic compounds have been studied for diagnosis of chronic lung allograft dysfunction (Kuppers et al., 2018), but to the date there are no studies assessing this test for IPA diagnosis.

### PTX3 and Cytokines

Other tests that have been explored by researchers to refine the diagnosis of IPA include the detection of biomarkers, such as Pentraxin-3 (PTX3) and cytokines. These tests are used in conjunction with standardized serological or other fungal diagnostic tests. One of these tests is monitoring the levels of PTX3. PTX3 is a soluble pattern recognition receptor, expressed after induction of inflammatory cytokines in response to inflammatory stimuli from endothelial cells and mononuclear phagocytes. Animal models showed PTX3 enhanced survival rate and reduced the lung fungal burden of infected rats (Lo Giudice et al., 2010). In a 2014 study in the stem cell transplant population, genetic deficiency of PTX3 was associated with increased risk of IPA in hematopoietic stem cell transplantation (HSCT) (Cunha et al., 2014). While not specific for *Aspergillus* spp., PTX3 has been used as diagnostic tool and for therapeutic monitoring in stem cell transplant studies (Biagi et al., 2008), where PTX3 levels were high in patients with IPA and decreased after treatment. However, only one group has explored this biomarker in the LTR population (Kabbani et al., 2017), where it was noted that PTX3 levels were significantly higher in BAL samples of LTRs with IPA. Patients with high levels of PTX3 in the BAL with positive GM or positive *Aspergillus* culture were 4.5 and 5.5 times more likely to have invasive pulmonary aspergillosis, respectively.

Identification of a specific signature of cytokines for IPA may be of tremendous help in the diagnosis of IPA. Cytokines have been studied more in depth in hematological malignancies; however, in these studies, a noted association has been made that higher levels of interleukin 10 (IL-10) were found in BAL of patients who also had IPA (Cunha et al., 2017) or worst outcomes in patients with persistently elevated IL-6 and IL-8 (Chai et al., 2010). To date, there haven’t been any studies assessing the role of cytokines in IPA in LTRs exclusively. One study that included LTRs among other solid organ transplant (SOT) recipients and hematological malignancies concluded that IL-1β, IL-6, IL-8, IL-17A, IL-23, and tumor necrosis factor alpha (TNFα) levels in BAL were significantly increased among patients with IPA, with IL-8 being the best marker (Gonçalves et al., 2017).

### MALDI-TOF

The incorporation of matrix-assisted laser desorption/ionization time-of-flight (MALDI-TOF) mass spectrometry to microbiology laboratories has been a revolution in the bacterial diagnosis of infections. Efforts are being made to apply this technique to other fields such as mycology. If implemented, this form of mass spectrometry will be a great advance in reducing the time of diagnosis. Yet a positive test will not be able to differentiate between colonization and disease in LTRs (Sanguinetti and Posteraro, 2014).

### TAFC Triacetylfusarinine C

Triacetylfusarinine C (TAFC) is a siderophore secreted by *A. fumigatus* after conidiospore germination in media where iron is scarce. Its synthesis is required for fungal germination, and it is crucial for the virulence factor of *A. fumigatus* in a mice models of IPA (Schrettl et al., 2004). Studies performed in patients with systemic erythematous lupus have proved that TAFC can be measured in the serum of these patients (Carroll et al., 2016). In immunocompromized patients it has been tested in a small cohort of patients with hematological malignancies, where it was found to increase the sensitivity of BAL GM (Orasch et al., 2017). Limitations of this test include a lack of larger studies, and the fact that it is mainly produced by *A. fumigatus* and *A. nidulans* (Charlang et al., 1981), meaning that some other species of *Aspergillus* that are also known to be etiological for IPA are excluded. To date there have been no studies in LTRs.

### Radiology

Radiology represents one of the pillars of IPA diagnosis. Most guidelines agree that while not specific to IPA alone, the typical radiological presentations in IPA include nodules, air crescent sign, cavity and halo sign. To complicate matters, however, the radiological presentation of IPA may be different in LTRs as compared to hematological malignancies or other SOT recipients. A 2014 study showed that bilateral bronchial wall thickening and centrilobular opacities with a tree-in-bud pattern were the most common radiological findings in a cohort of LTRs (Gazzoni et al., 2014). Another study that included LTRs among other SOT patients, found that halo sign was only observed in 8% of SOT recipients, whereas peri-bronchial consolidations were observed in 31% and ground-glass opacities in 38% (Park et al., 2010).

Positron emission tomography (PET) scans have also been studied for the diagnosis of IPA (Hot et al., 2011), but have a limited utility due to false positive results in patients with post-transplant lymphoproliferative disease (PTLD) or lung cancer (Baxter et al., 2011), and therefore must be used with caution. To avoid false positive results, immunological tests combined with diagnostic imaging such as PET scans or magnetic resonance imaging (MRI) are being developed. Using the monoclonal antibody JF5 combined with PET scan, researchers have found that the antibody binds to the antigenic determinant β1,5-galactofuranose present in a diagnostic antigen that is released by the pathogen during invasive growth in the lung (Davies et al., 2017). This strategy overcomes the problems of specificity faced by a computed tomography (CT) scans of the chest or MRI alone, or with the false positive results seen with the PET scan alone. However, the availability of this developing tool will be a limiting factor for its widespread use.

### Proposed Algorithm

**Figure 1:**
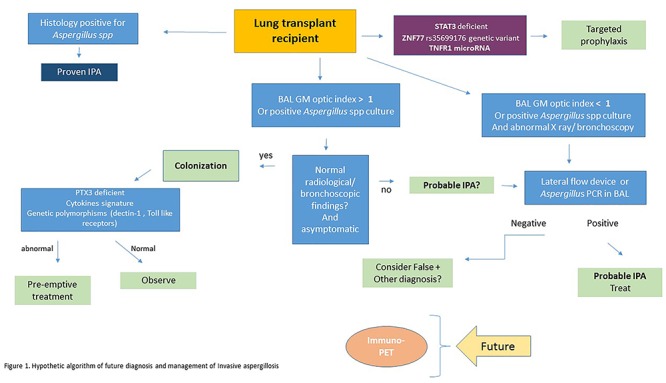
Hypothetic algorithm of future diagnosis and management of invasive aspergillosis.

Figure 1 shows a proposed algorithm for the future management and diagnosis of IPA. This algorithm includes some of the newer and developing diagnostic tools. One of the emerging factors that will likely play a large role in the future, is the detection of biomarkers related to a patient’s susceptibility to the disease, and the host’s potential to control angioinvasion. This will also be a determining factor in changing the landscape of how we manage prophylaxis of these patients, as it can help tailor a more individualized approach to treatment.

## Conclusion

### What the Future Holds

Despite the advances in transplantation and treatment of IPA, the development of an accurate diagnostic or prognostic tool for IPA continues to be a struggle.

New areas of research include identification of genetic susceptibility markers and prognostic indicators of IPA. One of those markers is Signal transducer and activator of transcription 3 (STAT3), a member of the STAT protein family and an important transcription activator has been found to have an important role in invasive fungal infections (Puel et al., 2011) as it is associated to the T-helper cell 17 (Th17) pathway (Yang et al., 2007). Inhibition of STAT3 had been shown to impair hyphal killing (Taylor et al., 2016) in animal models. Autosomal-dominant *STAT3*-deficient hyper-IgE syndrome is associated to susceptibility of late-onset mold infections, in particular *Aspergillus* (Vinh et al., 2010). STAT3 may hold the key to the timing when *Aspergillus* hyphae stops being a bystander, and starts to become an invasive disease.

Another set of biomarkers that can stratify the risk and prognosis of IPA is microRNA. MicroRNAs are non-coding RNAs capable of influencing gene expression through a variety of mechanisms (Lu and Rothenberg, 2013). Recent studies have examined MicroRNAs and identified those responsible for the regulation genes involved in the pulmonary immune responses following sub chronic inhalation exposure to *A. fumigatus* (Croston et al., 2016). Identification of microRNA’s regulation of, and impact on target genes might be able to assist in the diagnosis and treatment of IPA. Recently, zinc finger protein-77 (ZNF77) has been identified as a key player in *Aspergillus* colonization, by causing a loss of integrity of the bronchial epithelium and an increase in the levels of extracellular matrix proteins (Gago et al., 2018). These changes promote *A. fumigatus* conidial adhesion, germination and growth. However, these data have so far only been examined in limited sample sizes and need to be scrutinized in a larger cohort.

Lastly, there is an omnipresent need to perform larger studies in the LTR population. As transplant numbers continue to rise, larger studies would allow an improved comprehension of many of the tests described above which so far, have only been studied in small cohorts.

## Author Contributions

All authors contributed to the writing and revision of this manuscript.

## Conflict of Interest Statement

The authors declare that the research was conducted in the absence of any commercial or financial relationships that could be construed as a potential conflict of interest.

## Abbreviations

BAL: bronchoalveolar lavage
BG: beta-glucan
CF: cystic fibrosis
CMV: cytomegalovirus
COPD: chronic obstructive pulmonary disease
CT: computed tomography
ELISA: enzyme-linked immunosorbent assay
EORTC: European Organisation for Research and Treatment of Cancer
GM: galactomannan
HSCT: hematopoietic stem cell transplantation
IL: interleukin
IPA: invasive pulmonary aspergillosis
ISHLT: international society for heart and lung transplantation
LAL: *Limulus* amebocyte lysate
LTRs: lung transplant recipients
MALDI-TOF: matrix-assisted laser desorption/ionization time-of-flight
MRI: magnetic resonance imaging
PCR: polymerase chain reaction
PET: positron emission tomography
PTLD: post-transplantation lymphoproliferative disease
PTX3: pentraxin-3
SOT: solid organ transplant
STAT3: signal transducer and activator of transcription 3
TAFC: triacetylfusarinine C
Th17: T-helper cell 17
TNFα: tumor necrosis factor alpha
ZNF77: zinc finger protein-77
